# Social support in older adults: Validation and norm values of a brief form of the Perceived Social Support Questionnaire (F-SozU K-6)

**DOI:** 10.1371/journal.pone.0299467

**Published:** 2024-03-19

**Authors:** Anna C. Reinwarth, Julia Petersen, Manfred E. Beutel, Martin Hautzinger, Elmar Brähler

**Affiliations:** 1 Department of Psychosomatic Medicine and Psychotherapy, University Medical Center of the Johannes Gutenberg-University Mainz, Mainz, Germany; 2 Department of Psychiatry and Psychotherapy, University Medical Center of the Johannes Gutenberg-University Mainz, Mainz, Germany; 3 Department of Psychology, Clinical Psychology and Psychotherapy, Eberhard Karls University, Tübingen, Germany; 4 Department of Psychiatry and Psychotherapy, Medical Faculty, University of Leipzig, Leipzig, Germany; Second Xiangya Hospital, Central South University, CHINA

## Abstract

**Background:**

Social relations are crucial for maintaining physical and mental health across the life span. As social roles, networks and needs change with age a valid assessment of social support in older adults and age-specific norms are necessary. The present work aims to [1) assess the level of social support in individuals > 60 years of the general German population, [2) evaluate the brief six-item form of the Perceived Social Support Questionnaire (F-SozU K-6) in this age group and to [3) provide age-specific norm values.

**Methods:**

We analyze data of *N* = 706 people representative for the German population collected in 2021. To assess social support, we used the F-SozU K-6. We tested for selectivity, item difficulty, internal consistency, construct and factor validity, as well as factorial invariance. Additionally, we assessed correlations and associations with depression, loneliness, and sociodemographic factors. Furthermore, we reported norm values for respondents > 60 years.

**Results:**

Participants > 60 years reported a mean level of 23.97 (*SD* = 4.82) of social support. Results of the CFA confirmed a very good model fit. Measurement invariance across sex and age was shown. Associations with ADS and LS-S supported construct validity. Multiple regression analysis showed that female sex, increasing age, having a partner, and a higher equivalized household income were associated with higher levels of social support.

**Conclusion:**

The F-SozU K-6 is a reliable and economical tool to assess perceived social support in older adults. Norm values for individual > 60 years are provided.

## Introduction

For human beings, a social species, social relations are crucial to ensure safety, reproductive success and survival. From an evolutionary perspective, social relations with a mate or a tribe improve the chances of survival in hostile environments [1]. Indeed, the essential role of social relations in maintaining physical and mental health has been confirmed by several empirical studies [e.g., 2–5]. A recent meta-analytic review showed that individuals’ social experiences predict mortality comparably or even more strongly than well-established risk factors (e.g., obesity, smoking, alcohol consumption) [2]. Furthermore, social support is recognized as an important protective factor in preventing suicidal ideation [6, 7].

Critical life events (retirement, loss of spouse, family members, and friends, moving into a nursing home, worsening health, and functional decline), which affect the extent of social integration, social needs and social roles, become more prevalent with age. International organizations (e.g., the World Health Organization) had recognized social isolation as a significant social and policy concern in aging [8]. As recently shown by a systematic review and meta-analysis, older adults risk for social isolation is shaped by a complex interplay of biological factors, socioeconomic factors, psychological and behavioural factors [9]. Despite being female, having low education level or being divorces, low social support is a commonly known risk factor for being social isolated in older adulthood [10], To date a multitude of studies also highlighted the relevance of perceived social support in maintaining well-being, physical and mental health in the second half of life. For instance, a systematic review analysing the role of social support for the development and course of coronary heart diseases, showed an impact of low social support on the prevalence of coronary heart disease and pointed to the consistently found negative effect of low social support on cardiac and all-cause mortality [11]. Further, low social support were associated with acute and chronic elevations in blood pressure and heart rate [12]. A representative study of US community-dwelling older adults, found that persons who reported a lack of social support indicated poorer health status compared to persons who were satisfied with the social support available to them, which in turn is a main predictor of mortality [13]. Social support was also found to moderate the relationship between frailty and depression in older persons aged ≥60. With increasing social support, the negative effect of physical frailty on depressive symptoms was attenuated [14]. Empirical findings also suggested that social support may be an important target for intervention efforts for cognitive decline and dementia [15]. A systematic review, which analysed the impact of social activities, social networks and social support on cognitive function of adults aged ≥ 50 years, showed a positive relation of social support and global cognition and episodic memory [16]. Further, results of a recent scoping review including eleven meta-analyses and systematic reviews of social connections as possible determinants of cognitive decline in older adults with or at risk of developing Alzheimer’s disease and related dementias, pointed to the importance of social engagement and social activities in reducing risk of cognitive decline [17]. Even in late adulthood the motivation to enhance one’s status and esteem through work and contributions to a larger social network remains important [18].

The association of social relations and health can be explained by two theoretical approaches: the stress buffering [19] hypothesis and the main effects model [20]. The buffering hypothesis proposes indirect effects of social relations. Social relations provide informational, emotional, or tangible resources which in turn promote adaptation in case of acute or chronic stress. The deleterious effects of stressors on health are moderated or buffered by social resources. In contrast, the main effects model suggested more direct cognitive, emotional, behavioral or biological effects of social relations on health by stimulating brain activity, providing meaningful roles and promoting health and self-care.

To date, a multitude of different definitions and measurement approaches of social relations exist in literature. However, three main aspects are distinguished consistently across studies: the degree of social integration in social networks, supportive interactions or received social support and subjective beliefs and perception of available support also described as perceived social support. While the degree of social integration represents structural aspects of social relations, received and perceived social support represents functional aspects [20].

In an aging population with increasing health care needs, there is an urgent need to assess social support in the older population. Therefore, economic and validated self-assessment measures are needed, especially for large scale surveys. In the last decades, many scales were developed to assess social support. Most of these are unsuited for use in clinical and epidemiologic studies due to their large number of items (e.g., Duke-UNC Functional Social Support Questionnaire [21], Social Provisions Scale [22] or Social Support Questionnaire [23]). However, available short forms often showed low validity (e.g., short form of the Social Provision Scale [22]) or have not been psychometrically evaluated in the general population (e.g., short form of the Social Support Questionnaire [24]). The Social Support Questionnaire (F-SozU) [25, 26] and its short versions (F-SozU K-22 [27], F-SozU K-14 [28], F-SozU K-6 [29]) are widely used and valid measures to assess perceived social support in German-speaking general populations. The F-SozU was originally developed by Sommer & Fydrich [26]. The authors operationalized social support in functional manner as perceived or anticipated support from the social network. This cognitive approach was suggested by Cobb [30] and focuses on one’s subjective beliefs and perception of available support from the social network. The aspect of perceived social support attains higher significance in clinical and epidemiologic contexts compared to formal or structural aspects of social support [27]. The F-SozU captured three central dimensions of perceived social support via three subscales among 54 items: instrumental support (e.g., receiving practical advice), emotional support (e.g., being liked and accepted by others), and social integration (e.g., knowing people with similar interests). These subdimensions can be combined to a total score of the extent of one’s general perceived or anticipated social support. In line with the original 54-item version of the F-SozU, the 22-item version covers also the three central dimensions of perceived or anticipated social support. The 14-item version of the F-SozU also containing statements of all three dimensions, but focuses exclusively on general perceived social support without further distinction of instrumental, emotional or social integration aspects. Therefore, an unidimensional interpretation of the resulting total score was suggested for the F-SozU K-14. The F-SozU K-6 is the latest and shortest version. The Scale was developed, evaluated and standardized by Kliem, Mossle [29] based on representative German population samples. The F-SozU K-6 covers various aspects of perceived social support and showed good reliability compared to the longer versions (F-SozU K-22, F-SozU K-14). For the F-SozU K-14 as well as the F-SozU K- 6, an unidimensional interpretation of a total score was suggested. In accordance with previous forms of the F-SozU, perceived social support measured with the F-SozU K-6 showed negative correlations with depression, anxiety, and somatic symptom strain [29].

To date, the F-SozU K-6 is an implemented instrument for a short and reliable assessment of perceived social support in national and international research. Within a representative German community sample social support was considered as an important social component of resilience using the F-SozU K-6. Low social support also statistically predicted somatic symptoms and was associated with distress and adverse childhood experiences [31]. Especially, as the debate about the impact of the public health measures of social distancing on mental health and loneliness increased during the COVID-19 pandemic [32, 33], a multitude of studies analysing the mental health consequences of the pandemic have used the F-SozU K-6. For instance, an adapted version was used by Sommerlad, Marston [34], who reported that higher perceived social support was associated with lower symptoms of depression during COVID-19 lockdown in UK residents. Further, a study analysing predictors of mental health during the early months of the pandemic in the U.S. showed negative associations of social support with depression, anxiety and mental stress [35]. Furthermore, a recent cross-culturally psychometric evaluation of the F-SozuU K-6 attested its validity in Chinese, German, Russian, and U.S. samples [36]. Just as previously reported by Kliem, Mossle [29], the authors found negative correlations with depression, anxiety, and stress measures across the culture-samples. Measurement invariance across the samples accounting for its usefulness within cross-cultural epidemiologic studies [36]. However, a validation of the brief form (F-SozU K-6) in older adults is still lacking.

## Methods

### Aim

The purpose of the present work was to provide a validation of the F-SozU K-6 in individuals over 60 years of the general German population. Specifically, we aimed to:

1. assess the prevalence and determinants of social support in participants > 60 years
2. determine the psychometric properties (item characteristics, reliability, factorial structure, invariance across sex and age, construct validity) of the brief six-item form of the Perceived Social Support Questionnaire F-SozU K-6 in this age group,
3. provide norm values for older adults according to sex and age group (≤ 70 vs. ≥ 71) of a representative sample of the German population.

### Study design and participants

For this particular investigation, we utilized data from a nationwide, representative German population survey conducted by the independent market research institute USUMA. This survey started on 02 October 2021 and ended on 09 December 2021. It aimed to be representative in terms of age, sex, and educational level. The selection of participating households followed a random route procedure combined with Kish selection [37]. Prior to data collection, participants were provided with detailed information about the study’s procedures, data collection, and anonymization of personal data. Verbal informed consent was obtained as participants were ask to fill out a questionnaire and acknowledged by trained interviewers of USUMA. Before questionnaire were handed out, sociodemographic information of the participants was conducted in a personal interview. Then, the participants were ask to complete the questionnaire independently and to return it to the interviewers in a sealed envelope. While participants filling out the questionnaire, interviewers were not in the room but could help with comprehension problems. Finally, the previously assessed sociodemographic information was linked to the completed questionnaire without names or other identifying information. The study contents and procedures, including the consent procedure were approved by the institutional ethics review board of the University Leipzig (numbers: 474/20ek). The survey also adhered to the guidelines outlines in the ICC/ESOMAR International Code of Marketing and Social Research Practice in addition to ICH-GCP-guidelines and was conducted by the Declaration of Helsinki.

For our analysis we created a subsample, including only respondents over the age of 60 years. Within these subsamples, we also excluded participants with missing data for the FSozU K-6. Sample characteristics of the analysis sample with *N* = 706 participants are depicted in Table 1. The sample included 49.3% women and 50.7% men. A small number of participants who did not identify as either male or female was excluded from the analyses (*N* = 3). Mean age of the participants was 71.14 (*SD* = 7.18).

**Table 1:** Sample characteristics of the participants.

	All (*N* = 706)	
	*N*	*%*
**Sociodemographic information**		
Sex		
*male*	358	50.7
*female*	348	49.3
Age (*M*, *SD*)	71.14 (7.18)	
Age group		
*≤ 70 years old*	377	53.4
*> 70 years old*	329	46.6
Education		
*High school degree*	109	15.4
*No high school degree*	597	84.6
Employment		
*Employment (fulltime or part-time)*	106	15.0
*No paid employment*	599	85.0
Equivalized household income		
*< 1250€*	182	25.9
*1250–2500€*	460	65.4
*> = 2500€*	61	8.7
Partnership		
*With partner*	362	51.3
*Without partner*	344	48.7
Marital status		
*Married/ living together*	327	46.3
*Married/ not living together*	17	2.4
*Unmarried*	55	7.8
*Divorced*	119	16.9
*Widowed*	188	26.6
Number of persons in household (*M*, *SD*)	1.53 (0.56)	
**Psychological measures**		
Social support (F-SozU K-6; *M*, *SD*)	23.97 (4.82)	
Depression (ADS; *M*, *SD*)	12.42 (9.34)	
Loneliness (LS-S; *M*, *SD*)	3.23 (2.84)	

*Note*. *M* = Mean; *SD* = Standard deviation.

### Measures

#### Sociodemographic characteristics

Age, sex (male/female) identified by interviewers, employment status, and having a partner were surveyed. School education was assessed by asking about the highest degree achieved and recoded in 0 (no high school diploma) and 1 (having a high school diploma). Finally, household income was surveyed using the following income categories: 1 = under 500€, 2 = 500–650€, 3 = 650–750€, 4 = 750–900€, 5 = 900–1000€, 6 = 1000–1150€, 7 = 1150–1250€, 8 = 1250–1500€, 9 = 1500–2000€, 10 = 2000–2500€, 11 = 2500–3500€, 12 = 3500–5000€, 13 = over 5000€. In order to calculate the equivalized household income, we first assigned each respondent the mean value of the group they reported to be in. Then we divided this value by the root from the number of people living in the respondents’ household.

#### Social support

Social support was measured using the brief form of the Perceived Social Support Questionnaire (F-SozU K-6) [29]. This inventory comprises six items, which covers general aspects perceived social support, e.g., “There is someone very close to me whose help I can always count on” or “I know several people with whom I like to do things”. Participants rated each item on a five- point Likert scale, ranging from 1 (does not apply) to 5 (exactly applicable). Answers are summarized to a sum score (5–30), with higher scores indicating higher levels of perceived social support. In the present sample, the F-SozU K-6 showed a very good internal consistency (ω = 0.93).

#### Depression

Depression symptoms were assessed with the *Allgemeine Depressionsskala* (General Depression Scale, ADS) [38]. The ADS comprises 20 items (including four inverse items that were inverted prior to forming the sum score) on the frequency of typical depressive affective, cognitive, somatic and social symptoms and starts with the statement: “During the past week, everything was exhausting for me". Each item is scored on a scale from 0 (never or less than one day) to 3 (mostly or all the time). Answers are summarized to a sum score (0–60), with higher score indicating higher levels of depressive symptoms. In the present sample, the ADS showed a very good internal consistency (ω = 0.93).

#### Loneliness

Loneliness was captured by the Loneliness Scale-SOEP (LS-S) [39]. The LS-S assesses absence of companionship, rejection by peer groups, and feelings of social isolation as basic aspects of the subjective experience of loneliness. On a five-point Likert scale ranging from 0 (never) to 4 (very often), participants indicated how often the feeling of absence of companionship, rejection by peer groups, and social isolation occurred. Answers are summarized to a sum score (0–12), with higher scores indicating higher levels of loneliness. In a representative German population sample, the LS-S has previously shown good internal consistency and measurement invariance for sex and age [40]. In our sample, we found, like other scientists, the LS-S scale to have good internal consistency (ω = 0.89).

### Statistical analysis

Descriptive characteristics of the analysed sample were reported as absolute numbers and percentages for categorical variables and as means with standard deviations for continuous variables.

In order to assess selectivity, we computed the correlation of each specific F-SozU K-6 item with the total sum of all other items, resulting in item-total correlations. To determine the item difficulty, we summed the values obtained and divided them by the sum of the maximum attainable item values. Internal consistency of the score was determined via McDonald’s Omega (ω).

We tested factor validity using a confirmatory factor analysis (CFA), in which all items loaded on one factor. We used maximum likelihood estimation since we found significant deviations from normal distribution. This approach is a robust method in dealing with the violation of normality [41]. Furthermore, we assessed the goodness-of-fit of this model, based on four criteria: the Standardized Root Mean Squared Residual (SRMR), the Root Mean Square Error of Approximation (RMSEA), the Comparative Fit Index (CFI), and the Tucker Lewis Index (TLI). A model with a good fit would demonstrate a RMSEA and SRMR of < 0.050, with values between 0.050 and 0.080 indicating a reasonable fit, and a CFI and TLI of or >0.950 for a good fit [42, 43].

Additionally, we tested for factorial invariance, first across sex and then age groups. For these tests, we were guided by Meredith and Teresi’s [44] sequential strategy, in which we evaluated the configural, the weak, the strong, as well as the strict invariance of our model. Configural invariance is based on the assumption that there might be variations in the loadings, intercepts, and variances of the latent constructs among the different groups. Weak measurement invariance constrains factor loadings to be equal across all groups. Strong measurement invariance takes it a step further by constraining both factor loadings and item intercepts to be equal across the groups. Lastly, strict measurement invariance goes even further by mandating equality not only in factor loadings and intercepts but also in residual variances among groups. In this measurement invariance assessment, each model is compared against the more stringent model. The commonly used method for assessing overall model fit is the chi-square test. However, this test’s accuracy is influenced by sample size, potentially leasing to the rejection of reasonable models in cases of large sample sizes. To address this limitation, we rely on the abovementioned four fit indices in order to compare model fits. We can assume that a tested measurement in given if the fit of the more constrained model, primarily assessed through the CFI as proposed by Chen [45] is not significantly worse than the CFI of the less constrained model. This is the case if the CFI difference between the models does not surpass the 0.01 cut-off [45–47].

To test construct validity, we investigated intercorrelations of the brief form (F-SozU K-6) with LS-S and ADS. Intercorrelations were analysed as Spearman correlations. Further, we tested group differences for the F-SozU-6 score between the age groups (61–70 vs. 71+), sex (male vs. female), educational degrees (high school degree vs. no high school degree), having a partner (yes vs. no), and being currently unemployed (yes vs. no) using t-tests. Additionally, we reported effect sizes using Cohen’s d. Further, we tested the associations between these variables and the F-SozU in a multiple regression analysis.

We used percentiles to calculate normative values for the F-SozU-6 from the population over 60. Those percentiles were calculated for the total sample and for subsamples based on age. For all data analyses, we used the software R version 4.1.2 and RStudio version 2021.09.2 (packages: dplyr [48], psych [49], lavaan [50]).

## Results

### Prevalence and determinants social support

In 2021, participants reported a mean level of 23.97 (*SD* = 4.82) of social support. Higher levels of social support were found in women (*M* = 24.22; *SD* = 4.85) compared to men (*M* = 23.73; *SD* = 4.85).

### Item characteristics

Table 2 shows means (M) and standard deviations (SD) for the items as well as item difficulties (P_i_) and corrected item-total correlation **(*r***_***it***_**)**. In the total sample, participants scored highest on the item “There is someone very close to me whose help I can always count on” and lowest on the item “I receive a lot of understanding and security from others”. The item difficulties varied between 0.72 and 0.83.

**Table 2:** Means (M), standard deviations (SD), item difficulties (*P*_*i*_), corrected item-total correlations (*r*_*it*_), and group differences for the F-SozU K-6 items.

Item			total				With partner				Without partner				Group difference	
	English	German	M	SD	*P*_*i*_	*r*_*it*_	M	SD	*P*_*i*_	*r*_*it*_	M	SD	*P*_*i*_	*r*_*it*_	*t*	*p*
1	I receive a lot of understanding and security from others.	Ich erfahre von anderen viel Verständnis und Geborgenheit.	3.64	0.95	0.72	0.68	3.80	0.81	0.76	0.57	3.48	1.04	0.70	0.73	-4.515	**0.000**
2	There is someone very close to me whose help I can always count on.	Ich habe einen sehr vertrauten Menschen, mit dessen Hilfe ich immer rechnen kann.	4.14	0.97	0.83	0.78	4.41	0.75	0.88	0.65	3.88	1.08	0.78	0.82	-7.607	**0.000**
3	If I need to, I can borrow something from friends or neighbors without any problems.	Bei Bedarf kann ich mir ohne Probleme bei Freunden oder Nachbarn etwas ausleihen.	4.08	0.92	0.82	0.70	4.20	0.82	0.84	0.69	3.96	0.99	0.79	0.69	-3.501	**0.000**
4	I know several people with whom I like to do things.	Ich kenne mehrere Menschen, mit denen ich gerne etwas unternehme.	3.99	0.97	0.80	0.69	4.15	0.85	0.83	0.67	3.83	1.06	0.77	0.68	-4.556	**0.000**
5	When I am sick, I can ask friends/relatives to handle important things for me without hesitation.	Wenn ich krank bin, kann ich ohne Zögern Freunde/Angehörige bitten, wichtige Dinge für mich zu erledigen.	4.11	0.94	0.82	0.83	4.32	0.79	0.86	0.78	3.92	1.03	0.78	0.84	-5.743	**0.000**
6	If I’m very depressed, I know who I can turn to.	Wenn ich mal sehr bedrückt bin, weiß ich, zu wem ich damit ohne weiteres gehen kann.	4.01	1.07	0.80	0.79	4.30	0.87	0.86	0.76	3.74	1.17	0.75	0.79	-7.186	**0.000**

In the total sample, the corrected item-total correlations achieved very satisfactory values of *r*_*it*_ = 0.68 to *r*_*it*_ = 0.83. The corrected item-total correlations in participants with and without a partner can be regarded as satisfactory with values between *r*_*it*_ = 0.57 and *r*_*it*_ = 0.78 in participants with a partner and *r*_*it*_ = 0.68 and *r*_*it*_ = 0.84 in participants without a partner.

### Internal consistency

Including the total sample, we calculated a high internal consistency of ω = 0.93 for F-SozU K-6.

### Factor validity and factorial invariance

Results of the CFA confirmed a very good model fit. All assessed fit indices indicated an adequate to very good model fit with the exception of the RMSEA which was greater than 0.08 (CFI = .966; TLI = .943; SRMR = 0.030; RMSEA = 0.119, 90% confidence interval [0.093–0.147]). Factor loadings of all items were high (0.70 to 0.89). Though the RMSEA surpasses the proposed cut-off of 0.080, Byrne (2004) and Byrne and Stewart (2006) [51, 52] have suggested to mostly use the CFI as a fit indicator since it is more robust and less susceptible to larger samples. Since the CFI yields a good result, we assume our model to have a good fit.

We then analysed the measurement invariance regarding sex and age as depicted in S1 Table. Strict invariances can be assumed according to the CFI differences, which were all well below the cutoff-value of D_CFI_ = 0.01 as proposed by Chen [45].

### Construct validity

We found low but substantial intercorrelations of the F-SozU K-6 with LS-S and ADS in the expected directions, indicating its construct validity. As depicted in Table 3, higher perceived social support was associated with lower depression (ADS) and lower levels of loneliness (LS-S).

**Table 3:** Correlation coefficients between the F-SoZu K-6 and other self-rating questionnaires.

Fragebogen	F-SOZU K-6	ADS	LS-S
F-SOZU K-6	1.00		
ADS	-0.46***	1.00	
LS-S	-0.43***	0.54***	1.00

*Note*. Abbreviations: ADS = General Depression Scale, LS-S = Loneliness Scale-SOEP; Spearman’s correlation coefficient was used

***p < 0.001

Reported social support differed between participants with and without a partner. Participant with a partner had a higher average score of social support. We found no differences in social support between age groups, males and females, educational degrees, and employed and unemployed participants. For details see Table 4.

**Table 4:** Group differences in perceived social support (F-SozU K-6) of the participants.

	N	M	SD	N	M	SD	t	p	d
age (61–70 vs. 70+)	377	23.81	5.05	329	24.15	4.54	-0.93	0.350	0.071
sex (male vs. female)	358	23.73	4.79	348	24.22	4.85	-1.37	0.170	0.102
education (high school degree vs. no high school degree)	109	23.88	4.79	597	24.50	5.01	-1.20	0.234	0.125
partner (yes vs. no)	344	25.19	3.87	362	22.82	5.33	-6.78	**0.000**	-0.507
unemployment (yes vs. no)	599	23.88	4.77	106	24.49	5.13	1.13	0.259	0.126

*Note*. *N* = size of the (partial) sample; *M* = mean; *SD* = standard deviation; separate *N*, *M*, and *SD* for group 1 or group 2; *t* = t-value; *p* = p-Value; *d* = effect size (Cohen’s *d*); varying *N* due to partially missing measured values. “Unemployment” in our case means that the individual does not pursue any paid work, it therefore includes unpaid civil service work, parental leave, and retirement.

We additionally performed a multiple regression analysis with sex, age, education, partnership, equivalized household income and unemployment as explanatory variables. The results can be viewed in Table 5. Female sex, increasing age, having a partner, and a higher equivalized household income were positively associated with the F-SozU.

**Table 5:** Multiple regression analysis with perceived social support (F-SozU K-6) as outcome (*N* = 702).

*Model fit*:	F(7,694) = 11.352, p = 0.000; R^2^ = 0.103; R^2^_adj._ = 0.094			
	B	SE	95% CI	p
Sex (ref. male)	1.346	0.364	0.617–2.074	**< 0.001**
Age	0.077	0.027	0.020–0.133	**0.008**
Education (Abitur, yes)	0.320	0.511	-0.727–1.366	0.549
Partner (yes)	2.764	0.363	2.037–3.490	**< 0.001**
Equivalized household income (ref. < 1250€)				
1250–2500	1.341	0.411	0.484–2.198	**0.002**
> 2500	1.786	0.744	0.277–3.296	**0.020**
Unemployment (yes)	-0.829	0.555	-2.016–0.358	0.171

*Note*. *B* = unstandardized coefficient; *SE* = standard error; *CI* = confidence interval; *p* = p-value. Significant results marked in bold. Varying *N* compared to full sample due to missingness in explanatory variables.

### Norm values

Table 6 presents the norm values, additionally stratified by sex and age. Because the F-SozU K-6 sum score deviated from normal distribution, we opted to report the score distribution using percentiles. Here we can see a slight tendency of older respondents to report higher levels of social support, as we have already shown in the regression analysis.

**Table 6:** Percentiles for the F-SozU K-6 according to sex and age.

		Men		Women	
F-SozU K-6 mean	All	≤ 70	> 71	≤ 70	> 71
	*N* = 706	*N* = 194	*N* = 164	*N* = 183	*N =* 165
1	0	1	0	0	0
1.5	1	1	0	1	1
1.67	1	2	1	1	1
1.83	2	2	2	2	1
2	3	4	4	4	2
2.17	4	4	4	4	2
2.33	8	6	5	5	3
2.5	6	7	8	7	4
2.67	8	9	9	9	5
2.83	11	12	11	14	7
3	15	16	15	21	8
3.17	17	18	16	22	11
3.33	21	22	21	25	14
3.5	25	24	27	29	21
3.67	31	30	32	34	26
3.83	39	39	40	40	36
4	52	52	55	52	49
4.17	57	58	61	55	56
4.33	65	66	69	63	60
4.5	72	73	79	69	67
4.67	79	80	86	75	75
4.83	89	91	95	86	82
5	100	100	100	100	100

*Note*. Normative data are presented as F-SozU K-6 mean scores with corresponding percentiles. Percentiles are shown for the total sample and for subsamples based on sex and age.

## Discussion

The Social Support Questionnaire (F-SozU) and its short versions (F-SozU K-22, F-SozU K-14, F-SozU K-6) are widely used and valid measures to assess perceived social support. However, a validation of the shortest version F-SozU K-6 in older adults has been lacking to date. The present work aimed to validate and standardized the F-SozU K-6 and to report norm values for older adults of a German representative population sample.

Participants reported high levels of social support. We found item characteristics and the reliability of the F-SozU K-6 to be satisfactory. Results of the CFA confirmed the previous proposed [29] unidimensional interpretation of a total score among adults older than 60 years. Thus, F-SozU K-6 appears to be a reliable and economical tool to assess perceived social support in older adults. We additionally tested for measurement invariance across sex and age groups. Both models yielded good model fits confirming strict measurement invariance which indicates the ability to compare those groups. Though the RMSEA was slightly higher than typically acceptable in most models, we argue that this does not impact our findings, since the CFA–which showed good results and only small changes between the models–is the more reliable fit index [51, 52].

In accordance with previous research [e.g., 29, 31, 35] we found negative correlations with depression and loneliness, supporting its construct validity. Moreover, a regression analysis showed, clear positive associations between female sex, increasing age, having a partner, and having a higher equivalized household income with social support. These results were partly comparable to previously reported associations of sociodemographic characteristics and the longer version F-SozU K-14 in participants between 14 and 92 years [28]. Thus, we replicate the positive association of having a partner with social support in our sample of participants aged > 60 years. In contrast to Fydrich, Sommer [28], women reported more social support compared to men even beyond the age of 60 years. Further, we were not able to support the negative association of increasing age and social support in our sample of participants older than 60 years like it previous studies found. It could be assumed that living conditions and opportunities for participation in older age have improved in recent years accounting for these differences. Additionally, the onset on the pandemic might also have had an influence on our results: Previous study from Germany have found that, contrary to expectations, the population that suffered the most under the lockdown restrictions were not old people, but younger people. This somewhat surprising finding may be due to numerous factors such as loss of childcare for young families and the subsequent double burden of taking care of their children while simultaneously working. Other stressful factors for younger people might have been loss of income, youth culture, and social exchange that came with the closure of schools, universities, and entertainment sources [53, 54]. These factors may have affected the daily routines of the elderly less. Additionally, the elderly might have experienced prior crises that might have increased their resilient coping skills. However, older people oftentimes suffer from infrequent possibilities for social participation due to critical life events such as retirement, loss of spouse, family members, and friends, moving into a nursing home, worsening health, and functional decline. At the beginning of the pandemic, older people were identified as potential risk groups not only for the virus but for increased loneliness. It is possible that this increased the public’s awareness for their loneliness risk and has motivated the relatives of this older population to increase contact to them through more frequent calls or visits, especially as soon as they were allowed again. Subsequently, their amount of social interaction might have actually even increased during the pandemic compared to pre-pandemic levels, decreasing their loneliness levels and potentially increasing their perception of their social support levels. In contrast, younger people who had a more established social network pre-pandemic, might have suffered more since their social interactions were drastically limited due to lockdowns. This implies that policy makers and healthcare providers should (a) make an effort in identifying potential risk groups of loneliness and low social support by assessing them individually, (b) report these findings to the public, increasing awareness to this risk, and (c) establish measures to garner social support for these risk groups, especially in times like a global pandemic. As research shows, this is especially important since low social support has been linked to acute and chronic elevations in blood pressure and heart rate, a generally poorer health status, the relationship between frailty and depression, and all-cause mortality [11–14]. Social support was also found to possibly be an important target for intervention efforts for cognitive decline and dementia [15]. Potential measures could be, for example, promoting group activities to increase the social network of the individuals and counselling for those that feel like they have a low level of support. Furthermore, we recommend future research on determinants of social support in participants aged > 60 years to identify important influencing factors and vulnerable subgroups. In line with Otten, Ernst [6], we found that higher levels of social support were associated with a higher equivalized household income.

We also reported norm values across sex and age groups (61–70, 71+). These norm values, again, indicate higher levels of social support in older age.

### Limitations

Despite the great strength of the present study using data of a representative German population survey, results should be interpreted with respect to study’s limitations. While our findings are representative for older adults of the German general population, comparisons can only be made for Western demographics. Thus, we recommend replication of our results within similar studies performed by nations with other cultural backgrounds. Psychometric properties may also change when the scale is applied to different samples, e.g., clinical population of older adults. Furthermore, the survey from which we drew our data were conducted during the COVID-19 pandemic while severe restrictions on physical social contact (e.g., working from home, restrictions regarding the number of non-household members allowed to meet) were implemented in Germany. These restrictions might pose unique challenges to social support exchanges and increased the risk of social isolation. A recent empirical investigation suggested that, the perceived quality of social relations was worse during the pandemic compared to before. Therefore, we cannot exclude potential effects of the COVID-19 pandemic and its accompanying measures on the reported perceived social support within our study, as we have discussed.

## Conclusion

Psychometric indicators, e.g. reliability of the F-SozU K-6 were very satisfactory. The factor analysis confirmed the unidimensional factorial structure while measurement invariance could be confirmed across age and sex. Results from a multiple regression analysis show that women, individuals with a higher age, a partner and a higher equivalized household income were more likely to have higher levels of social support. These findings indicate a need for assistance of singles, men, as well as individuals from lower socioeconomic backgrounds since social support was also significantly associated with lower levels of depression and loneliness. Going from there, the scale might be also used to evaluate whether an intervention had an effect on social support or not. Summed up, the F-SozU K-6 can be used to as a reliable and valid screening tool to identify vulnerable subgroups and those likely to benefit most from social support interventions among older adults.

## Supporting information

S1 Table Fit indices for testing for measurement invariance with a model for the total sample and models grouped by gender and age. (DOCX)

S2 Table Brief form of the Perceived Social Support Questionnaire (F-SozU K-6). (DOCX)
