# Supplementary material for: Social support in older adults: Validation and norm values of a brief form of the Perceived Social Support Questionnaire (F-SozU K-6)
Source: PLoS One. 2024 Mar 19;19(3):e0299467. doi: 10.1371/journal.pone.0299467 (PMC10950225; doi:10.1371/journal.pone.0299467)
Supplement: S1 Table — (DOCX) [file pone.0299467.s001.docx]

| **total sample** | | | | | | | | |
| --- | --- | --- | --- | --- | --- | --- | --- | --- |
|  | **CFI** | **TLI** | **RMSEA** | **SRMR** | **Factor loadings** |  |  |  |
| Configural | 0.966 | 0.943 | 0.119  CI [0.093 – 0.147] | 0.030 | 0.71  0.84  0.72  0.70  0.89  0.86 |  |  |  |
| **grouped by gender** | | | | | | | | |
|  | **CFI** | **TLI** | **RMSEA** | **SRMR** | △ **CFI** | △ **TLI** | △ **RMSEA** | △ **SRMR** |
| Configural | 0.959 | 0.931 | 0.132 | 0.032 |  |  |  |  |
| Weak | 0.957 | 0.944 | 0.118 | 0.045 | -0.002 | 0.013 | -0.014 | 0.013 |
| Strong | 0.956 | 0.953 | 0.109 | 0.047 | -0.001 | 0.009 | -0.009 | 0.002 |
| Strict | 0.955 | 0.961 | 0.100 | 0.050 | -0.001 | 0.008 | -0.009 | 0.003 |
| **grouped by age (60-75 / 75+)** | | | | | | | | |
|  | **CFI** | **TLI** | **RMSEA** | **SRMR** | △ **CFI** | △ **TLI** | △ **RMSEA** | △ **SRMR** |
| Configural | 0.964 | 0.941 | 0.122 | 0.032 |  |  |  |  |
| Weak | 0.965 | 0.955 | 0.106 | 0.034 | 0.001 | 0.014 | -0.016 | 0.002 |
| Strong | 0.960 | 0.957 | 0.104 | 0.040 | -0.004 | 0.002 | -0.002 | 0.006 |
| Strict | 0.955 | 0.960 | 0.100 | 0.047 | -0.005 | 0.003 | -0.004 | 0.007 |
