# Supplementary material for: Social support in older adults: Validation and norm values of a brief form of the Perceived Social Support Questionnaire (F-SozU K-6)
Source: PLoS One. 2024 Mar 19;19(3):e0299467. doi: 10.1371/journal.pone.0299467 (PMC10950225; doi:10.1371/journal.pone.0299467)
Supplement: S2 Table — (DOCX) [file pone.0299467.s002.docx]

| Kreuzen Sie bitte das Kästchen an, das Ihrer Zustimmung am besten entspricht. Wenn in den folgenden Aussahen allgemein von „Menschen“ oder „Freunden/Angehörigen“ die Rede ist, dann sind die Menschen gemeint, die Ihnen wichtig sind. | | | | | |
| --- | --- | --- | --- | --- | --- |
|  | Trifft nicht zu | Trifft eher nicht zu | Trifft teilweise zu | Trifft zu | Trifft genau zu |
| Ich erfahre von anderen viel Verständnis und Geborgenheit. |  |  |  |  |  |
| Ich habe einen sehr vertrauten Menschen, mit dessen Hilfe ich immer rechnen kann. |  |  |  |  |  |
| Bei Bedarf kann ich mir ohne Probleme bei Freunden oder Nachbarn etwas ausleihen. |  |  |  |  |  |
| Ich kenne mehrere Menschen, mit denen ich gerne etwas unternehme. |  |  |  |  |  |
| Wenn ich krank bin, kann ich ohne Zögern Freunde/Angehörige bitten, wichtige Dinge für mich zu erledigen. |  |  |  |  |  |
| Wenn ich mal sehr bedrückt bin, weiß ich, zu wem ich damit ohne weiteres gehen kann. |  |  |  |  |  |

Kliem S, Mossle T, Rehbein F, Hellmann DF, Zenger M, Brahler E. A brief form of the Perceived Social Support Questionnaire (F-SozU) was developed, validated, and standardized. J Clin Epidemiol. 2015;68(5):551-62.
